# Awareness and willingness to use pre-exposure prophylaxis for HIV prevention among men who have sex with men in Rwanda: findings from a web-based survey

**DOI:** 10.3389/fpubh.2024.1325029

**Published:** 2024-03-01

**Authors:** Athanase Munyaneza, Viraj V. Patel, Nataly Rios Gutierrez, Qiuhu Shi, Benjamin Muhoza, Gallican Kubwimana, Jonathan Ross, Etienne Nsereko, Gad Murenzi, Laetitia Nyirazinyoye, Leon Mutesa, Kathryn Anastos, Adebola Adedimeji

**Affiliations:** ^1^Research for Development (RD Rwanda), Kigali, Rwanda; ^2^School of Public Health, College of Medicine and Health Sciences, University of Rwanda, Kigali, Rwanda; ^3^Division of General Internal Medicine, Montefiore Medical Center, Bronx, NY, United States; ^4^New York Medical College, Valhalla, NY, United States; ^5^Center for Human Genetics, College of Medicine and Health Sciences, University of Rwanda, Kigali, Rwanda; ^6^Department of Epidemiology and Population Health, Albert Einstein College of Medicine, Bronx, NY, United States

**Keywords:** men who have sex with men, pre-exposure prophylaxis, web-based survey, Rwanda, sub-Sahara Africa

## Abstract

**Introduction:**

Pre-exposure Prophylaxis (PrEP) is a daily pill aimed at reducing HIV transmission risk when taken as prescribed. It’s highly recommended for high-risk Men who have sex with Men (MSM). This study aimed to assess PrEP awareness and willingness to use it among Rwandan MSM, a critical aspect given PrEP’s proven effectiveness. The findings are expected to inform policy decisions and further advance the implementation of PrEP strategies.

**Methods:**

This is a cross-sectional study design that utilized a web-based survey conducted between April and June 2019 to assess awareness and willingness to use PrEP among sexually active MSM in Rwanda. A snowball sampling technique was used to recruit participants via social media such as WhatsApp and e-mail. Eligibility criteria included being sexually active, aged ≥18 years, self-identifying as MSM, residing in Rwanda, self-reported engagement in receptive or insertive anal sex in the last 12 months, and self-reported HIV-negative serostatus. We assessed two primary outcomes: PrEP awareness (having ever heard of PrEP) and willingness to use PrEP within one month of completing the survey. Multivariable logistic regression was performed to identify participant characteristics associated with PrEP awareness and willingness to use it.

**Results:**

Out of 521 participants, the majority (73%) demonstrated awareness of PrEP. Factors linked to PrEP awareness included residing outside the capital, Kigali, being in the 18–29 age group, having higher education levels, perceiving a benefit from PrEP, and engaging in vaginal sex with a woman while using a condom in the last year. Additionally, 96% of participants expressed a strong willingness to use PrEP.

**Conclusion:**

Rwandan MSM exhibits a high level of PrEP awareness, notably associated with factors like location, age, education, perceived benefits, and condom use. The study also revealed a strong willingness to use PrEP, indicating promising prospects for its adoption among this group. These findings highlight the need for targeted awareness campaigns, personalized interventions, and comprehensive sexual health education to promote PrEP adoption and strengthen HIV prevention efforts among Rwandan MSM.

## Introduction

Pre-exposure prophylaxis (PrEP) is highly effective in preventing new Human Immunodeficiency Virus (HIV) infections (1) and is recommended by the World Health Organization (WHO) as a key prevention strategy (2, 3). Sexual and gender minority (SGM) men who have sex with men (MSM) in sub-Saharan Africa (SSA) are disproportionately impacted by HIV, with an average HIV prevalence of 17% (range 3.7–33.4%) (4, 5) r as compared to 6% (range:0.5 to 19.7%) of men in general population (6). The prevalence of HIV among MSM in SSA is influenced by the legal and social environment, where in some countries homosexuality is considered a crime attracting severe penalties (7).

The Integrated Biological and Behavioral Surveillance Survey (IBBSS) conducted in 2020 revealed that the overall HIV prevalence among MSM in Rwanda was 4.3%, with the highest rate in Kigali City at 11.3% (5). Furthermore, a study conducted in the same year among MSM and transgender women in Kigali reported an HIV prevalence of 10% (8). This indicates that the prevalence of HIV infection among MSM in Rwanda is two to three times higher than in the general population (8–10) thus necessitating additional HIV preventive interventions in this vulnerable group. Rwanda has recognized PrEP as a crucial element of its HIV prevention strategy (11). However, despite a country-wide rollout of PrEP in 2019 (12), there is limited information on its awareness, willingness to use it, access to it, and its utilization among key populations like MSM.

Awareness of PrEP among MSM varies between countries due to different economic statuses and supporting policies (13). In SSA, PrEP awareness among MSM is generally relatively low, ranging from 20 to 60% (9, 13–15) compared to other key populations. PrEP awareness varies by demographic characteristics such as education, age, and migration status (16). Willingness to use PrEP increases over time (17), but varies substantially by country, ranging from 32 to 92% (17), suggesting a variety of factors associated with the likelihood of using it. Despite this, there are encouraging signs of increasing awareness as more African countries implement PrEP projects (18), and use appears to be increasing. Example is that In Rwanda, the PrEP-to-need ratio is >1, indicating positive progress. However, challenges such as limited availability (19), and access (20) persist, underscoring the need for data on factors determining awareness of and willingness to use PrEP among at-risk MSM (21).

A Kenyan study revealed that individual and interpersonal factors such as condom use self-efficacy, perceived ability to use PrEP, and membership in a Lesbian, Gay, Bisexual, Transgender (LGBT) organization, contributed significantly to the awareness of and willingness to use PrEP (22). We previously described awareness of and willingness to use PrEP in MSM in Rwanda, reporting that nearly half (48%) of MSM sampled for the study were aware of PrEP, and most (83%) were willing to use it (9). However, the sample was limited to a cohort of MSM living in the City of Kigali, therefore, it was not representative of MSM in the country in Rwanda, and therefore a comprehensive survey was required to include MSM residing outside of Kigali. The objective of the present study using an online survey was to assess awareness of and willingness to use PrEP among a more representative population of Rwandan MSM. Highlighting these aspects can guide efforts to enhance awareness and accessibility to PrEP in this vulnerable population.

## Methods

### Study design, setting, and population

We conducted a cross-sectional study in Rwanda using an anonymous online survey between April and June 2019. Ethics approval for the study was granted by the Rwanda National Ethics Committee and the Albert Einstein College of Medicine Institutional Review Board. We collaborated with leaders from MSM organizations in Kigali who participated in prior research (9, 21) to form a Community Advisory Board (CAB) for the study. MSM organizations focus on specific human rights issues for members of their community and, in collaboration with various partners, provide HIV and Sexually Transmitted Infection (STI) testing and prevention support and linkage to care for members of their community who need those services. The CAB facilitated the testing and validation of the study questionnaire, provided information about the study to participants, and supported the distribution of the survey link to community members as needed.

Eligibility criteria for participants included: age ≥ 18 years, individuals whose sex at birth was male and self-identify as MSM, residence in Rwanda, self-reported engagement in receptive or insertive anal sex in the last 12 months, and self-reported HIV-negative serostatus. We excluded participants from a prior and ongoing cohort studies t because of their pre-exposure to PrEP-related information during the study visits (23). The language of the survey questionnaire was Kinyarwanda, which is widely spoken in Rwanda.

### Data collection

**Figure 1:**
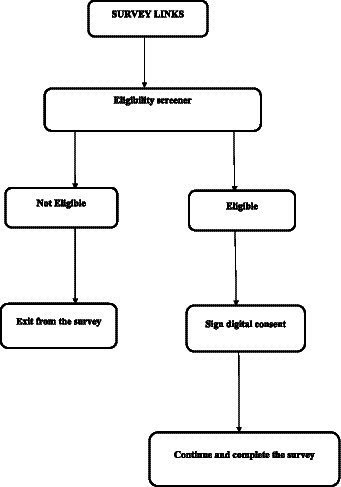
Process for participating in the study survey.

The questionnaire was piloted with members of the CAB and subsequently revised before distribution to eligible participants. Using Qualtrics (24), web-links generated were sent through WhatsApp or by e-mail. It was requested that CAB members disseminate the survey web URLs to individuals within their social network and to members of their organizations. Upon obtaining the survey link, participants were requested to respond to the screening survey questions in order to ascertain their eligibility. If found to be eligible, they proceeded to complete a digitized informed consent form prior to commencing the survey (Figure 1). In addition, the survey was distributed by the participants who responded, as the last question asked them to share the survey link with their friends and social networks that they knew or felt were MSM. Participants who completed the survey were given an incentive of 2,500 Rwandan francs (~$2), which they received through mobile money transfers to their cell phones.

### Outcomes and measures

We assessed two main outcomes, (1) awareness of PrEP, which was preceded by an introductory text as follows: “PrEP is a new way to prevent getting infected with HIV. PrEP is a pill taken once a day by an HIV-negative person to protect themselves from getting HIV BEFORE they have sex. You can stop using the medicine at any time, in consultation with a doctor/nurse/healthcare worker. PrEP is extremely effective and safe.” Following this introduction, participants were asked: “Before today, how much would you say you knew about PrEP?” Responses were categorized as follows: “I have never heard of PrEP, I’ve heard about it, but I did not know what it is, I know a little bit about it, I know a fair amount about it, I know a lot about it.” (2) Willingness to use PrEP, by asking: “If PrEP were available for free, would you be willing to start taking PrEP in the next 1 month to protect yourself from getting HIV”? Responses were categorized as “yes, no, not sure.”

Independent variables included: (1) Socio-demographic characteristics including age (18–24, 25–29, 30 years and above), place of residence (Kigali, another place in Rwanda), highest educational level completed (no education, primary, secondary, vocational, and university) and income (no income, < 150, 000 RWF, and ≥ 150, 000 RWF), (2) General and sexual health status including time since last consultation with a healthcare provider (never, less than six months, six months to one year, and more than one year ago), willingness to receive information about sexual health or to stay health through social media was included, categorized as no versus yes, (3) Sexual behaviors including current relationship status (not in a relationship versus being in a relationship with a man, a woman or a transgender), disclosure of sexual identity (to friends and/or colleagues, family members, and healthcare providers), having anal sex without a condom with another man or woman in the last 12 months, (4) perceived benefit in taking PrEP by asking: “*Do you think you might benefit from PrEP*” with Yes versus No response options.

### Data analysis

Data were analyzed using IBM SPSS statistics (version 21). Responses for variables on PrEP awareness were dichotomized as “**Yes**” (“I’ve heard about it, but I did not know what it is, I know a little bit about it, I know a fair amount about it, I know a lot about it”) and, “**No**”(“I have never heard about PrEP”). For willingness to use PrEP, responses were dichotomized as either **Yes** or **No** (“no, not sure”). Educational level was analyzed as a three-category variable (no education or completed primary, completed secondary or vocational training, completed university).

We conducted the analysis in three stages. First, for the characterization and description of study participants, we generated frequencies and proportions from categorical variables. Next, we used bivariate logistic regression models to determine the statistical significance of the relationship between independent variables and study outcomes, calculating odds ratios (ORs) and 95% confidence intervals (CI). Finally, we conducted multivariable logistic regression analysis, including only independent variables with a value of *p* <0.05 in the bivariate models. The adjusted odd ratios (aOR) and 95% CIs were used to present the associations between independent and outcome variables.

### Data flow

**Figure 2:**
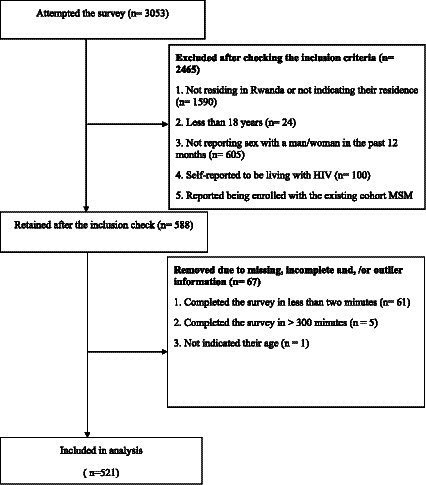
Data flow.

A total of 3,053 clicks were recorded for the survey, out of which 2,465 were excluded due to ineligibility. Additionally, 67 response clicks were excluded because respondents either completed the survey in an unusually short time (<2 min, 61 cases) or took an excessively long time (>300 min, 1 case) and because age information was not reported in 5 cases. Consequently, the final analytic sample comprised 521 participants (see Figure 2).

## Results

### Socio-demographic characteristics of participants

Among the 521 participants included in this analysis, 318 (61%) reported living in the City of Kigali in the past 6 months, while 203 (39%) resided outside of the City of Kigali during the same period. Furthermore, 330 of 521 (63%) participants were 24 years of age or younger. Additionally, 319 (62%) reported having completed either secondary or vocational education, while 379 (73%) indicated they had no monthly income (Table 1).

**Table 1:** Characteristics and descriptions of study participants.

Variable	Frequency	Percent
*Living area in the past 6 months*		
Kigali	318	61
Another place in Rwanda	203	39
*Age of respondents*		
18–24 years	330	63.3
25–29 years	141	27.1
30 years and above	50	9.6
*Highest level of education completed*		
None or primary	120	23.4
Secondary + vocational	319	62.3
University	73	14.3
*Monthly income*		
No income	379	73.3
< 150,000 RWF	125	24.2
≥ 150,000 RWF	13	2.5
*Most recent consultation with a healthcare provider*		
Never	23	4.4
Less than 6 Months	291	55.9
6 months to 1 year	131	25.1
More than 1 year ago	76	14.6
*Willingness to receive information for staying health on WhatsApp, Facebook Messenger, SMS, E-mail, or on another program on your mobile phone*		
Yes	483	94.7
No	27	5.3
*You think you might benefit from PrEP*		
Yes	471	91.1
No	46	8.9
Current relationship status		
In a relationship (man, female or transgender)	101	19.6
Not in relation with some one	415	80.4
*Disclosure of sexual attractions to friends and/or colleagues/family member or providers*		
Yes	74	35.1
No one knows	137	64.9
*Having anal sex without a condom with another man in the last 12 months*		
Yes	234	47.6
No	258	52.4
*Having sex intercourse without a condom with a woman in the last 12 months*		
Yes	218	43.9
No	279	56.1
*Willingness to receive information about sexual health on WhatsApp, Facebook Messenger, SMS, E-mail, or on another program on your mobile phone*		
Yes	479	93.4
No	34	6.6
Awareness of PrEP		
I know about PrEP	381	73.1
I have never heard of PrEP	140	26.9
Willingness to use PrEP		
Willing to use	498	95.6
Not willing to use	23	4.4

### General, sexual health, behavior, and acceptability of technology to receive health information

Over half (291/521) of the participants (56%) reported having had a health consultation with a healthcare provider in the six months prior to survey completion. Nearly all (483 or 95% and 479 or 93%) of the participants reported that they were willing to receive information about staying healthy or sexual health using Whatsapp, Facebook Messenger, SMS, e-mail or any other virtual method.

About 415/521 (80%) reported that they were not in any sexual relationship at the survey period. One hundred and thirty-seven respondents (65%), reported that they have not disclosed their sexual orientation to anyone. Condom use in the past 12 months was reported by 258 (52%) of those who had anal sex with other men and 279 (56%) of those who had vaginal sex. In addition, most participants 471 (91%) thought that PrEP could benefit them.

### Awareness of and willingness to use PrEP

Overall, 381 (73%) participants reported awareness of PrEP. In the bivariate analysis, awareness of PrEP was found to be significantly associated with various social demographic characteristics, general, sexual health, and behavior variables among participants (Table 2). In the multivariable logistic regression analysis, individuals who reported living outside Kigali were almost twice as likely to be aware of PrEP (aOR 2.36, 95% CI 1.40–3.96) compared with those living in the City of Kigali. PrEP awareness was significantly higher among participants aged 18–24 years (aOR 2.28, 95% CI 1.03–5.01) and 25–29 years (aOR 3.06, 95% CI 1.36–6.93) compared to those aged ≥30 years. Similarly, participants with secondary/vocational education (aOR 1.77, 95% CI 1.01–3.07) and university education (aOR 2.65, 95% CI 1.18–5.97) reported higher awareness of PrEP compared to those with no education or only primary education. Additionally, awareness of PrEP was significantly higher among participants who perceived a benefit from PrEP (aOR 9.52, 95% CI 4.28–21.22) compared to those who did not. Furthermore, those reporting vaginal sex with a condom were more aware of PrEP (aOR 1.82, 95% CI 1.14–2.91) than those reporting vaginal sex without a condom. This is described in Table 3. Overall, 498 (96%) of participants were willing to start taking PrEP in the next one month to protect themselves from getting HIV. It is important to note that there was no statistical difference in willingness to use PrEP between those who were aware of PrEP and those who were not, *p* = 0.134.

**Table 2:** Bivariate analysis of PrEP awareness among Rwandan MSM.

	Bivariate analysis		
	PrEP awareness		
Variable	Yes (%)	No (%)	OR (95% CI)
*Living area in the past 6 months*			
Kigali	213 (67%)	105 (33%)	Ref
Another place in Rwanda	168 (82.8%)	35 (17.2%)	**2.366 (1.535–3.647)**
*Age of respondents*			
18–24 years	243 (73.6%)	87 (26.4%)	**2.578 (1.406–4.728)**
25–29 years	112 (79.4%)	29 (20.6%)	**3.568 (1.790–7.100)**
30 years and above	26 (52%)	24 (48%)	Ref
*Highest level of education completed*			
No Education + primary	81 (67.5%)	39 (32.5%)	Ref
Secondary + vocational	239 (74.9%)	80 (25.1%)	1.438 (0.910–2.275)
University	56 (76.7%)	17 (23.3%)	1.586 (0.817–3.080)
*Monthly income*			
No income	273 (72%)	106 (28%)	Ref
< 150,000 RWF	97 (77.6%)	28 (22.4%)	1.345 (0.835–2.166)
≥ 150,000 RWF	8 (61.5%)	5 (38.5%)	0.621 (0.19–1.942)
*Most recent consultation with a healthcare provider*			
Never	15 (60.9%)	9 (39.1%)	1.194 (0.461–3.094)
Less than 6 Months	222 (76.3%)	69 (23.7%)	**2.469 (1.456–4.186)**
6 months to 1 year	102 (77.9%)	29 (22.1%)	**2.699 (1.456–4.983)**
More than 1 year ago	43 (56.6%)	33 (43.4%)	Ref
*Willingness to receive information for staying health on WhatsApp, Facebook Messenger, SMS, E-mail, or on another program on your mobile phone*			
Yes	359 (74.3%)	124 (25.7%)	Ref
No	16 (59.3%)	11 (40.7%)	0.502 (0.227–1.112)
*Think you might benefit from PrEP*			
Yes	366 (77.7%)	105 (22.3%)	**8.848 (4.494–17.422)**
No	13 (28.3%)	33 (71.7%)	Ref
*Current relationship status*			
In a relationship with a man, female or transgender	62 (61.4%)	39 (38.6%)	Ref
Not in relation with some one	**317 (76.4%)**	**98 (23.6%)**	**2.035 (1.284–3.224)**
*Disclosure of sexual attractions to friends and/or colleagues/family member or providers*			
Yes	46 (62.2%)	28 (37.8%)	Ref
No one knows	92 (67.2%)	45 (32.8%)	1.244 (0.690–2.244)
*Having anal sex without a condom with another man in the last 12 months*			
Yes	165 (70.5%)	69 (29.5%)	Ref
No	196 (76%)	62 (24%)	1.322 (0.886–1.973)
*Having sex intercourse without a condom with a woman in the last 12 months*			
Yes	148 (67.9%)	70 (32.1%)	Ref
No	218 (78.1%)	61 (21.9%)	**1.690 (1.31–2.526)**
*Willingness to receive information about sexual health on WhatsApp, Facebook Messenger, SMS, E-mail, or on another program on your mobile phone*			
Yes	355 (74.1%)	124 (25.9%)	Ref
No	22 (64.7%)	12 (35.3%)	0.640 (0.308–1.332)

OR, Odd ratios; Ref, Reference category; CI, Confidence interval. Values in bold in table that are statistically significant.

**Table 3:** Multivariable analysis of PrEP awareness among Rwandan MSM.

	Multivariable analysis			
	PrEP awareness			
Variable	aOR	95% C.I.		*p*-value
Living area in the past 6 months		Lower	Upper	
Kigali	Ref			
Another place in Rwanda	**2.359**	**1.404**	**3.963**	**0.001**
*Age of respondents*				
18–24 years	**2.281**	**1.038**	**5.014**	**0.04**
25–29 years	**3.067**	**1.358**	**6.926**	**0.007**
30 years and above	Ref			
*Highest level of education completed*				
None or primary	Ref			
Secondary + vocational	**1.764**	**1.015**	**3.067**	**0.044**
University	**2.658**	**1.184**	**5.966**	**0.018**
*Most recent consultation with a healthcare provider*				
Never	0.872	0.29	2.626	0.808
Less than 6 Months	1.605	0.842	3.06	0.151
6 months to 1 year	1.763	0.853	3.644	0.126
More than 1 year ago	Ref			
*Think you might benefit from PrEP*				
Yes	**9.529**	**4.279**	**21.22**	**< 0.0001**
No	Ref			
*Current relationship status*				
In a relationship with a man, female, or transgender	Ref			
Not in relation with some one	1.747	0.96	3.177	0.068
*Having sex intercourse without a condom with a woman in the last 12 months*				
Yes	Ref			
No	**1.826**	**1.145**	**2.91**	**0.011**

Values in bold in table that are statistically significant.

## Discussion

The anonymous online survey conducted among MSM in Rwanda revealed important findings related to the awareness and willingness to use PrEP as an HIV infection prevention method. We observed that majority of respondents were aware of PrEP, and nearly all respondents were willing to initiate it in the near future as a protective measure against HIV infection. Furthermore, a significant proportion of the participants mentioned having had recent medical consultations and exhibited a very high willingness toward obtaining health-related information via diverse online platforms.

While our study reported high PrEP awareness, it is important to note that awareness levels have varied in previous research conducted in low- and middle-income countries (LMICs) (13, 16, 24–28), including our previous study of MSM living in the City of Kigali (9). This variability can be attributed to factors such as the clarity of PrEP definition provided to participants, the timing of the survey compared to PrEP availability (11, 29), and the broader trends in PrEP awareness over time (16). Rwanda’s inclusion of PrEP in national HIV prevention guidelines in 2018 (11, 29) may explain the higher awareness in our study compared to previous publications.

A high level of PrEP awareness is an indication that public health campaigns aimed at HIV/AIDS prevention are working. Knowledgeable people are more likely to actively seek information, speak with medical professionals, and make informed choices, which could lead to an increase in the use of PrEP among populations that are at risk (30–32). This awareness also enables public health organizations to target educational campaigns effectively, directing resources to populations with lower awareness to promote equitable access to PrEP information and hence potential use.

Our results indicate that awareness of PrEP was higher among MSM who reported residing outside Kigali in the past 6 months than among those who reported living in Kigali City. This suggests that factors beyond awareness campaigns and information access, such as individual living independence, may influence PrEP awareness. Mobility among MSM across Rwanda, as observed in a prior report (33), may contribute to this difference. A study conducted in high-income settings, including Atlanta, Chicago, and New York City, similarly documented disparities in PrEP awareness associated with place of residence and geographic location (34). Contrary to our findings, a study conducted among gay, bisexual, and other men who have sex with men (GBMSM) in Nigeria, suggested that those living in urban areas may exhibit higher levels of awareness (35). Our results indicated a different trend. Further research and in-depth data analysis are imperative to comprehensively discern the underlying factors contributing to the disparity in PrEP awareness among MSM in various regions of Rwanda.

Additionally, our research revealed that awareness of PrEP were associated with younger ages and higher educational attainment, which is in accordance with Rwanda’s demographic trends (5, 9), and similar findings in SSA (24, 35–38). These results highlight the need for targeted outreach and education efforts tailored to different age groups, particularly younger individuals who may be more receptive to new prevention methods. In addition, our findings also suggest that public health education and awareness campaigns should consider the educational background of the target audience to effectively disseminate information about PrEP.

Furthermore, participants who perceived a benefit from PrEP demonstrated substantially awareness of it, highlighting the importance of emphasizing PrEP’s advantages and effectiveness in HIV prevention in public health campaigns. The perception of PrEP benefits has consistently been identified as a factor facilitating its potential use among MSM (26).

We observed a significant association between condom use during vaginal sex and PrEP awareness. Those reporting condom use were more aware of PrEP, suggesting a connection between safe sex practices and knowledge about PrEP. Individuals who use condoms may consistently already have higher awareness of HIV prevention methods, making them more receptive to information about PrEP. Also, healthcare providers or sexual health education programs may be more likely to discuss PrEP with individuals who prioritize safe sex practices, leading to increased awareness. This finding highlights the significance of comprehensive sexual health education and outreach initiatives that provide information on PrEP in addition to contraceptive use or other protective measures promotion. Similarly, in a neighboring country, Kenya, higher condom use and self-efficacy have been reported to be associated with PrEP awareness (15).

Finally, the study revealed a high level of willingness among respondents to use PrEP, indicating readiness for PrEP implementation among MSM in Rwanda, in line with broader trends observed among MSM in sub-Saharan Africa (14, 25, 39, 40).

### Limitations

The survey’s anonymity and the use of snowball sampling to gather respondents may have contaminated responses from some participants who lived in the same community as individuals who had previously participated in another cohort study testing PrEP awareness (9). Furthermore, the limited number of online-based studies conducted with MSM in SSA restricts the effective comparison of these results across the region. While we wished to explore further the sources of information for those who were aware of PrEP, it’s important to note that this was a limitation of our survey design, as this specific aspect was not included in the survey questions. Also, our survey did not collect information on the specific province of each respondent. Finally, the overwhelming willingness of nearly all respondents to use PrEP prevented us from conducting a bivariate or multivariate analysis to identify potential associations between participants’ socio-demographic, health, and/or sexual health characteristics and their observed willingness to use PrEP.

## Conclusion

The results of the anonymous online survey indicate a high level of awareness of PrEP among MSM, with around two-thirds being aware of PrEP. Significantly, the vast majority of participants indicated a readiness to commence PrEP use within the following month as a preventive measure against HIV. A wide range of socio-demographic variables were found to be correlated with PrEP awareness, such as place of residence, age, level of education, perceived benefit from PrEP, and condom use. This study provides crucial information regarding Rwandan MSM’s awareness of PrEP and propensity to use it. This underscores the importance of implementing focused awareness campaigns, individualized interventions, and comprehensive sexual health education in order to encourage the use of PrEP and prevent HIV infection among this demographic. Policy-makers should design and implement targeted PrEP awareness campaigns that take into account the demographic variations in awareness identified in the survey. Special emphasis should be placed on reaching older MSM individuals and those with lower levels of education to ensure equitable access to information about PrEP. Highlighting the advantages of PrEP in HIV prevention can encourage more individuals to consider and use it as a preventive measure.

## Data availability statement

The raw data supporting the conclusions of this article will be made available by the authors, without undue reservation.

## Ethics statement

The studies involving humans were approved by the Rwanda National Ethics Committee and the Albert Einstein College of Medicine Institutional Review Board. The studies were conducted in accordance with the local legislation and institutional requirements. The participants provided their written informed consent to participate in this study.

## Author contributions

AM: Formal analysis, Investigation, Methodology, Project administration, Writing – original draft, Writing – review & editing. VP: Conceptualization, Formal analysis, Funding acquisition, Writing – review & editing, Methodology, Validation, Visualization. NG: Data curation, Formal analysis, Software, Writing – original draft. QS: Formal analysis, Methodology, Writing – review & editing, Visualization. BM: Data curation, Software, Writing – review & editing. GK: Project administration, Resources, Supervision, Writing – review & editing. JR: Methodology, Validation, Visualization, Writing – review & editing. EN: Methodology, Supervision, Validation, Writing – review & editing. GM: Conceptualization, Funding acquisition, Methodology, Project administration, Supervision, Validation, Writing – review & editing. LN: Supervision, Validation, Writing – review & editing. LM: Funding acquisition, Methodology, Validation, Writing – review & editing, Supervision, Visualization. KA: Conceptualization, Funding acquisition, Resources, Validation, Writing – review & editing, Methodology. AA: Conceptualization, Funding acquisition, Methodology, Resources, Supervision, Validation, Visualization, Writing – original draft.
